# Rituximab ameliorated severe hearing loss in Cogan's syndrome: a case report

**DOI:** 10.1186/1750-1172-5-18

**Published:** 2010-06-16

**Authors:** Jelka G Orsoni, Bruno Laganà, Pierangela Rubino, Laura Zavota, Salvatore Bacciu, Paolo Mora

**Affiliations:** 1 Institute of Ophthalmology, University of Parma - Parma, Italy; 2 University "La Sapienza" 2nd Faculty, S. Andrea Hospital - Rome, Italy; 3 Department of Otolaryngology - Head and Neck Surgery, University of Parma - Parma, Italy

## Abstract

**Background:**

Rituximab is a monoclonal antibody inducing depletion of B lymphocytes and presently approved for the treatment of non-Hodgkin's lymphoma and rheumatoid arthritis. Here is the first report of the use of this drug in a case of Cogan's syndrome (CS).

**Case Presentation:**

a 25-year-old Italian woman was referred with conjunctival hyperaemia, interstitial keratitis, moderate bilateral sensorineural hearing loss accompanied by tinnitus, dizziness, nausea and vertigo, poorly responsive to oral and topical steroidal therapy. Diagnosis of typical CS was made. The administration of a combined immunosuppressive treatment resolved ocular inflammation, dizziness, nausea, and vertigo but gave little results in controlling progressive hearing loss. A noticeable improvement in hearing function was documented by pure tone audiometry after infusion of Rituximab.

**Discussion:**

in CS, hearing function is often the most difficult parameter to control with therapy. A positive effect of Rituximab on was observed in our case. The drug also allowed to significantly reduce the number of adjuvant immunosuppressive medications.

## Background

Rituximab is a chimeric human-mouse monoclonal antibody against lymphocyte CD20 surface antigen, and treatment induces depletion of B lymphocytes by various mechanisms. These antibodies are thought to act in vivo mostly through activation of antibody-dependent cell-mediated cytotoxicity and complement-dependent cytotoxicity, although direct growth inhibition and/or induction of apoptosis may also take place [1]. Rituximab is presently approved for the treatment of non-Hodgkin's lymphoma and rheumatoid arthritis. Here is the first report of the use of this drug in a case of severe Cogan's syndrome (CS).

## Case Presentation

**Figure 1:**
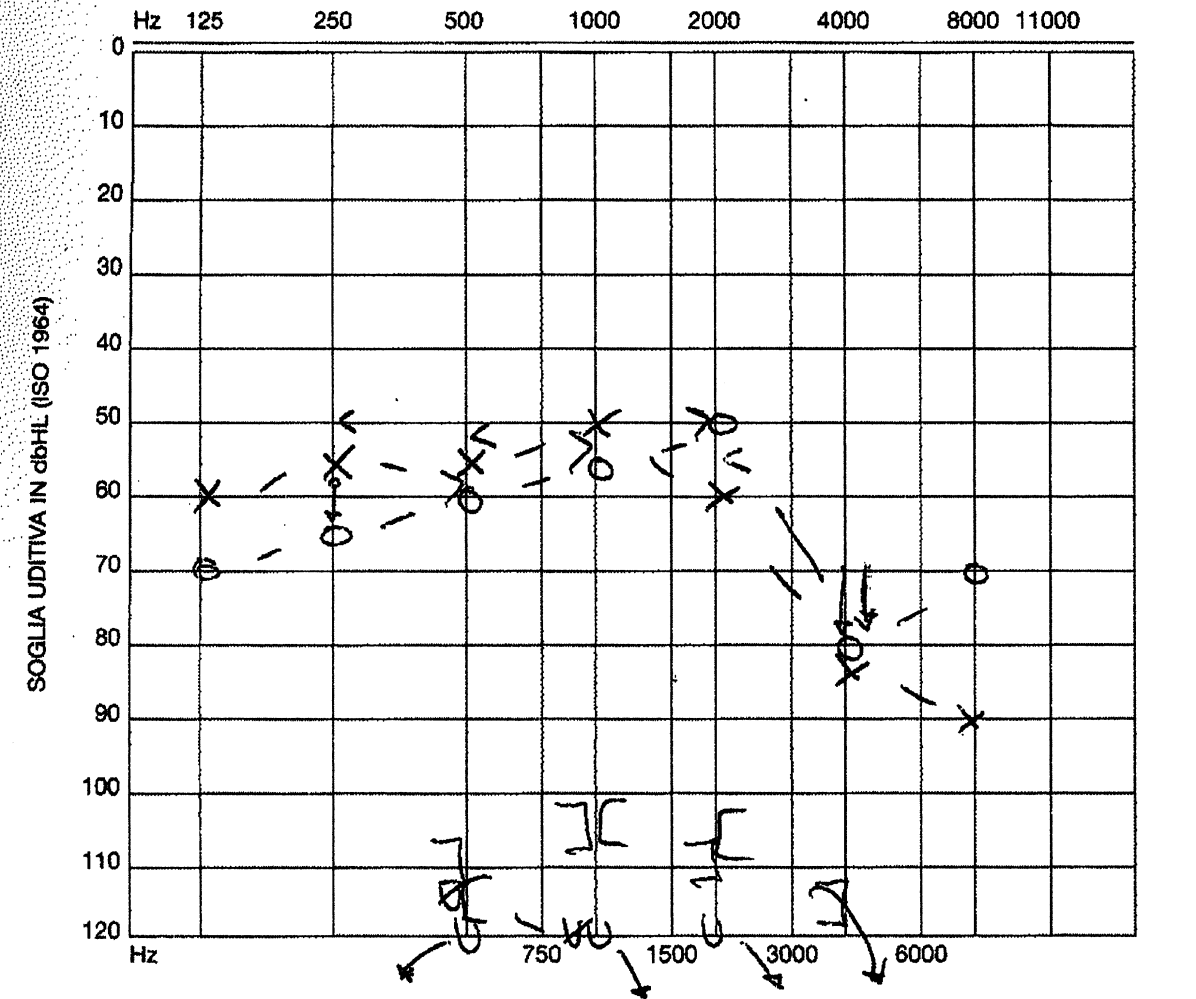
**The symbols "○" for the right ear and "x" for the left ear indicate the auditory threshold for a stimulus transmitted through the air of a frequency ranging from 125 to 800 MHz**. The symbols ">" for the right ear and "<" for the left ear represent the auditory threshold for a stimulus transmitted through the bone of a frequency ranging from 250 to 4000 MHz. The presented audiogram allows the calculation of the Pure Tone Average (PTA) for the central frequencies which quantifies the average auditory deficit as 61 dB for the right ear and 62 dB for the left ear. At the bottom of the figure the auditory thresholds of the stapedial reflex are shown.

**Figure 2:**
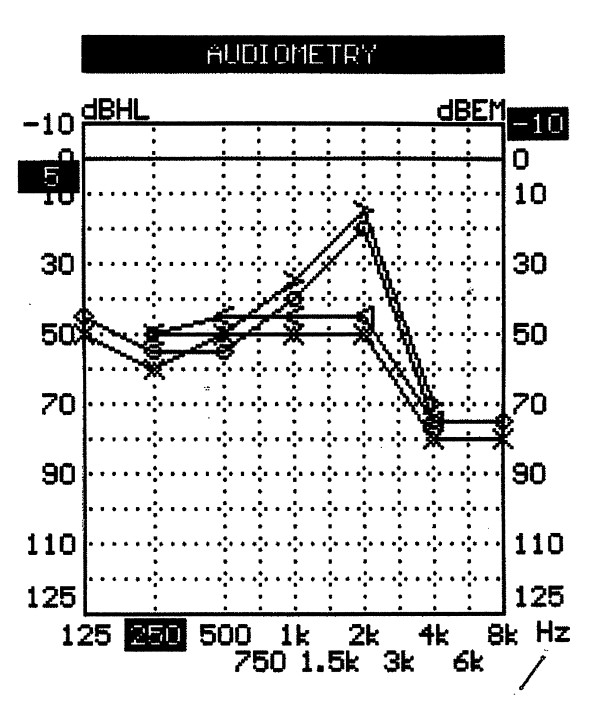
**The audiogram allows the calculation of the Pure Tone Average (PTA) for the central frequencies which quantifies the average auditory deficit as 45 dB (16 dB of improvement) for the right ear and 57 dB for the left ear**.

A 25-year-old Italian woman developed severe bilateral sensorineural hearing loss over the course of 12 months from her first acute inflammatory episode characterized by conjunctival hyperaemia, interstitial keratitis and tinnitus. Before the admission, the patient had already experienced a 3-week regimen of oral prednisone (50 mg/day at regressive dosage) and dexametasone eye-drops with poor response. After the patient had been screened for a possible infectious origin of her symptoms and all other known causes of interstitial keratitis had been excluded (i.e. syphilis, sarcoidosis, leprosy, Lyme disease, viruses, hypersensitivity to drugs), diagnosis of typical CS was confirmed by positive anti-hsp 70 antibody[2]. An immunosuppressive combination therapy was thus started following the reference protocol of our Centres for systemic autoimmune diseases[3]. It consisted of pulse intravenous cyclophosphamide (400 mg IV once a month, for 6 consecutive months), intramuscular methotrexate (10 mg/sqm once a week with folic acid integration), oral cyclosporine (2.5 mg/kg of lean body weight per day) and prednisone (40 mg per week). Treatment controlled the relapsing ocular inflammation and stopped progression of the typical, ring-shape interstitial keratitis. It was also successful in achieving good control of accompanying symptoms such as dizziness, nausea, and vertigo. The same treatment, however, gave poor results in controlling progressive hearing loss (figure 1). An anti-Tumour Necrosis Factor (anti-TNF) molecule (adalimumab, 40 mg once a week subcutaneously) was then associated to prednisone and methotrexate for 6 months, but hearing loss continued to worsen. Following the patient's informed consent and approval of local ethics committee, Rituximab was infused as an "off label" adjuvant rescue treatment at the dosage of 500 mg iv/week for 4 consecutive weeks. The same regimen was repeated after 6 months, while oral prednisone at 10 mg every other day was maintained. A noticeable improvement in hearing function was documented by pure tone audiometry starting from the 28^th ^day after the first drug infusion, mostly affecting the right ear (figure 2). The pre-treatment CD19+ lymphocyte count was 73 cells/uL, which then decreased below the detectibly threshold after the infusion cycles. No other undesirable effects were recorded during or after therapy. At 12 months of follow-up, the audiometry curve was still better than pre-treatment levels and essentially unchanged in comparison to that shown in figure 2. Other previously experienced manifestations of the syndrome remained under control.

## Conclusions

Cogan's Syndrome is a rare autoimmune vasculitis characterized by bilateral interstitial keratitis associated with vertigo, tinnitus and typically profound sensorineural deafness. This clinical presentation is called "typical" CS. "Atypical" CS is the name given to those forms with similar vestibuloauditory symptoms but ocular involvement different from interstitial keratitis (i.e. scleritis, retinal vasculitis, papillitis, etc), and/or other severe systemic vasculitic manifestation[4,5]. Considering both forms, CS is a rare clinical entity which usually occurs in early adulthood but with possible onset in children or in elderly patients [6,7]. Anti hsp-70 antibody has been indicated as a marker of the autoimmune origin of hearing loss [2].

While different immunomodulatory drugs, including mycophenolate and anti-TNF molecules, have been proposed in CS with satisfactory results, refractory cases are still reported, most concerning hearing function [7-10]. The use of Rituximab in systemic vasculitis and autoimmune diseases with antibody-mediated aetiology has a strong rationale and is increasingly reported in the literature [11,12]. In CS, hearing function seems to be the most difficult parameter to control with therapy. The effect of Rituximab on B cells may therefore be of interest to avoid deafness and the demand of cochlear implant in severe cases. It can also allow to significantly reduce the number of medications necessary to control the multiple manifestations of the syndrome. We recommend the four-week division of the overall cycle dose of the drug as it appears to be particularly safe, even though we do not recommend the use of this drug as a first line therapy.

## Competing interests

The authors declare that they have no competing interests.

## Consent

Written informed consent was obtained from the patient for publication of this case report and any accompanying images. A copy of the written consent is available for review by the Editor-in-Chief of this journal.

## Authors' contributions

All authors have read and approved the final manuscript. JGO, BL, SB and PM have made substantial contributions to conception and design, or acquisition of the data, or analysis and interpretation of the data. PR and LZ have been involved in drafting the manuscript and revising it critically for important intellectual content.
